# Measuring polar bear health using allostatic load

**DOI:** 10.1093/conphys/coaf013

**Published:** 2025-03-05

**Authors:** Sarah J Teman, Todd C Atwood, Sarah J Converse, Tricia L Fry, Kristin L Laidre

**Affiliations:** School of Aquatic and Fishery Sciences, University of Washington, 1122 NE Boat St Box 355020, Seattle, WA 98195, USA; U.S. Geological Survey, Alaska Science Center, 4210 University Drive, Anchorage, AK 99508, USA; US Geological Survey, Washington Cooperative Fish and Wildlife Research Unit, School of Environmental and Forest Sciences & School of Aquatic and Fishery Sciences, University of Washington, 1122 NE Boat St, Seattle WA 98105, USA; FWH Consulting, LLC, 4430 Yuma Dr., Madison, WI 53711, USA; School of Aquatic and Fishery Sciences, University of Washington, 1122 NE Boat St Box 355020, Seattle, WA 98195, USA; Polar Science Center, Applied Physics Laboratory, University of Washington, 1013 NE 40th St, Seattle, WA 98105, USA

**Keywords:** Allostatic load, Arctic, conservation physiology, marine mammal, polar bear*, Ursus maritimus*, wildlife health, **Abbreviations**: ALB = albumin; ALP = alkaline phosphatase; ALT = alanine aminotransferase; BAS = basophil count; BCI = body condition index; BUN = blood urea nitrogen; CA = calcium; CBC = complete blood count; CREA = creatinine; EOS = eosinophil count; GLOB = globulin; HCC = hair cortisol concentration; LYM = lymphocyte count; MON = monocyte count; NEU = neutrophil count; NA = sodium; PCV = packed cell volume; PHOS = phosphorus; POT = potassium; TP = total protein; UC ratio = urea–creatinine ratio

## Abstract

The southern Beaufort Sea polar bear sub-population (*Ursus maritimus*) has been adversely affected by climate change and loss of sea ice habitat. Even though the sub-population is likely decreasing, it remains difficult to link individual polar bear health and physiological change to sub-population effects. We developed an index of allostatic load, which represents potential physiological dysregulation. The allostatic load index included blood- and hair-based analytes measured in physically captured southern Beaufort bears in spring. We examined allostatic load in relation to bear body condition, age, terrestrial habitat use and, over time, for bear demographic groups. Overall, allostatic load had no relationship with body condition. However, allostatic load was higher in adult females without cubs that used terrestrial habitats the prior year, indicating potential physiological dysregulation with land use. Allostatic load declined with age in adult females without cubs. Sub-adult males demonstrated decreased allostatic load over time. Our study is one of the first attempts to develop a health scoring system for free-ranging polar bears, and our findings highlight the complexity of using allostatic load as an index of health in a wild species. Establishing links between individual bear health and population dynamics is important for advancing conservation efforts.

## Introduction

The polar bear (*Ursus maritimus*) is a sentinel Arctic species that has been impacted by the effects of climate warming and loss of sea ice habitat (U.S. Fish and Wildlife Service [USFWS], 2008). The species is listed as threatened under the US Endangered Species Act (USFWS, 2008) and Vulnerable by the International Union for the Conservation of Nature (IUCN) with an estimated circumpolar population of 26 000 individuals (Wiig *et al.,* 2015). Polar bears are distributed across 20 sub-populations that can differ geographically, genetically and behaviourally. One of the most well-studied sub-populations of polar bears is the southern Beaufort Sea (SB) sub-population, located in northern Alaska, USA, and northern Yukon and Northwest Territories, Canada. Abundance was most recently estimated for the Alaska portion of the SB sub-population at 565 bears (95% Bayesian credible interval [340, 920]; Bromaghin *et al.,* 2021), an estimated 25–50% decline (Bromaghin *et al.,* 2015) since monitoring began on this sub-population in the late 1960s (Amstrup *et al.,* 1986). Understanding SB bear health is important for conserving the sub-population and for maintaining its ecological role and its value as a subsistence and cultural resource for Iñupiat communities (e.g. Dowsley, 2010).

SB bears face multiple, cumulative threats to their health and survival because of a changing climate and an increasing human footprint in the Arctic (Atwood *et al.,* 2016a). Loss of sea ice habitat, which polar bears rely on as a platform to hunt their primary prey, ringed seals (*Pusa hispida*), has reduced hunting success and resulted in greater nutritional stress, poorer body condition (Rode *et al.,* 2010, 2014; Atwood *et al.,* 2021) and declines in breeding probabilities and recruitment rates (Regehr *et al.,* 2010; Rode *et al.,* 2010). In response to reduced foraging opportunities on the sea ice, some SB bears increasingly use terrestrial habitats in search of alternative or human-provisioned food resources (Schliebe *et al.,* 2008; Atwood *et al.,* 2016b; McKinney *et al.,* 2017). Greater use of terrestrial habitats by bears and expected northern expansion of pathogens due to climate change have increased the risk of exposure to a broader array of pathogens (Kirk *et al.,* 2010a; Atwood *et al.,* 2017). Infection from pathogens could increase physiological stress that may be further exacerbated by changes in diet or nutritional decline (Bowen *et al.,* 2015; Neuman-Lee *et al.,* 2017). SB bears exposed to contaminants, such as heavy metals or chlorinated hydrocarbons, may experience endocrine disruption (Routti *et al.,* 2019) or decreased immune signalling (Bourque *et al.,* 2020); these effects may be amplified during times of declining fat reserves (Atwood *et al.,* 2017; Routti *et al.,* 2019). The Beaufort Sea coastal plain in Alaska is home to the Arctic’s largest, and expanding, oil and gas industrial footprint (Houseknecht, 2019). The growing reliance on terrestrial habitat by bears has led to more frequent human–bear interactions and has raised concerns over exposure to anthropogenic noise that can increase physiological stress and disrupt bear behaviour (e.g. feeding or denning; Atwood *et al.,* 2016a, Larson *et al.,* 2020).

For decades, scientists have monitored the SB sub-population with nearly annual capture–mark–recapture efforts and health assessments, resulting in robust longitudinal datasets (Rode, 2020; Durner, 2021; Atwood, 2023). What remains elusive are the links between the health of individuals, individual fitness and sub-population-level outcomes (Patyk *et al.,* 2015). This knowledge gap is, in part, because polar bear carcasses are not reliably recovered (outside of subsistence-harvested animals) to allow for inference about causes of mortality, which could allow for an understanding of how health states of captured bears translate to types of mortality risks. Thus, it is difficult to identify when an individual bear is in a state of poor health, even in this well-studied sub-population.

Allostatic load shows promise to help to understand wildlife health and disease. Representing cumulative ‘wear and tear’ on the body, allostatic load refers to the physiological effects from chronic exposure to stress, including stress caused by social or environmental factors (McEwan and Stellar 1993). Decades of research on allostatic load in humans have shown that increased allostatic load can predict major morbidities and mortality (Seeman *et al.,* 1997; Parker *et al.,* 2022). Allostatic load has more recently been applied to investigations of wildlife health (Edes *et al.,* 2016; Edes and Crews 2017; Seeley *et al.,* 2022). A benefit of monitoring allostatic load is the ability to account for early signs of physiological dysregulation, i.e. incorporating transitionary measures of poor health that might not yet exceed the threshold of dysregulation, but are approaching it (Edes and Crews 2017). Although allostatic load can refer to explicit physiological dysregulation, we define it as the potential for physiological dysregulation, to account for uncertainty and variation in physiological states. In developing an allostatic load index for polar bears, we included analytes related to neuroendocrine stress, immune system activity, hydration, liver and kidney function and fasting status.

We present a novel allostatic load index for SB polar bears, and we evaluate the index as a reflection of potential physiological dysregulation by examining allostatic load in relation to a health outcome, onshore habitat use, bear age and time. We developed four different sets of hypotheses and associated predictions. First, we hypothesized that increased physiological stress is associated with poor health, and that allostatic load is indicative of a polar bear’s stress level. Following this, we predicted that a negative health outcome (e.g. decreased body condition) would be related to increased allostatic load in polar bears. Next, we hypothesized that polar bears using onshore habitat are exposed to greater numbers of pathogens and sources of anthropogenic stress, and so we predicted that allostatic load would be positively associated with onshore habitat use. We also evaluated two competing hypotheses regarding age: our first hypothesis posited that older bears have higher levels of physiological stress, and so we predicted a positive relationship between allostatic load and age. Our counter hypothesis suggested that younger, inexperienced individuals are more susceptible to stress, which would be supported by a negative relationship between allostatic load and age. Finally, we hypothesized that overall levels of physiological stress are increasing with climate change and an increasing human footprint in the SB, and so we predicted that we would see an increase in allostatic load over time.

## Materials and Methods

### Data collection

We used data collected from SB polar bears that were captured, sampled and released by the US Geological Survey between 1983 and 2018 (Rode, 2020; Durner, 2021; Atwood, 2023). Spring captures of polar bears typically occurred between 20 March and 5 May (Fry *et al.,* 2019). The SB study area was generally bounded by Utqiağvik (formerly Barrow), Alaska to the west, the US–Canada border to the east, Alaska’s coastline to the south, and ~90 km from the coast over the sea ice to the north (Fry *et al.,* 2019). Blood was collected from immobilized polar bears using previously described methods (see Fry *et al.,* 2019 and 2023). Serum biochemistry was analysed using the VetScan VS2 Biochemistry Analyser and included albumin (ALB; g/dl), alkaline phosphatase (ALP; U/I), alanine aminotransferase (ALT; U/I), blood urea nitrogen (BUN; mg/dl), calcium (CA; mg/dl), creatinine (CREA; mg/dl), globulin (GLOB; g/dl), sodium (NA; mmol/l), phosphorus (PHOS; mg/dl), potassium (POT; mmol/l) and total protein (TP; g/dl) (Fry *et al.,* 2019, 2023; Atwood, 2023). Complete blood count (CBC) with differential and packed cell volume (PCV) were obtained for captures between 2005 and 2018, via manual counts for captures before 2008 (Kirk *et al.,* 2010b) or via Abaxis HM5 analyser thereafter (Atwood, 2023; Fry *et al.,* 2023). These data included basophil count (BAS; count per microlitre), eosinophil count (EOS; count per microlitre), lymphocyte count (LYM; count per microlitre), monocyte count (MON; count per microlitre), neutrophil count (NEU; count per microlitre), and PCV. Hair was collected to measure hair cortisol concentration (HCC) (Durner, 2021); see Van der Walt *et al.* (2021) for methodology. Bears were aged (see Fry *et al.,* 2023 for methods), weighed (in kilogrammes) and measured for total length (in centimetres) (Rode, 2020). Total length was measured from the tip of the nose to the base of the tail prior to 2002, and henceforward, from the tip of the nose to the tip of the tail. Pre-2002 total length data were adjusted by adding mean tail length. Capture and handling procedures were conducted under proper permits, including Marine Mammal Research Permit MA690038–17 and US Geological Survey Animal Care and Use Committee approval 2017–03.

### Allostatic load index

- *Specifying the allostatic load index*

We defined the allostatic load index based on known physiological measures of polar bears, including analytes representing metabolic, immune or neuroendocrine function (Table 1). ‘Analyte’ includes traditional biomarkers for which the presence and quantity is indicative of some known biological process, such as HCC (representing a physiological stress response) or the urea–creatinine (UC) ratio (representing short-term fasting status in bears), as well as serum biochemical analytes or differential white blood cells that correlate with specific states. Metabolic analytes included those related to liver or kidney functioning (e.g. ALB, ALP, ALT, GLOB) or hydration status (e.g. ALB, NA, PCV) (Fry *et al.,* 2019, 2023). Metabolic analytes were also selected if they have a narrow biologically normal range (e.g. CA, PHOS, POT) that could reveal a state of dysregulation. Measures of immune response included BAS, EOS, LYM, MON and NEU. Neuroendocrine functioning was represented by the HCC.

**Table 1:** Analytes selected for inclusion in the allostatic load index for polar bears in the southern Beaufort Sea. This table is adapted from Table 1 in Fry *et al.* (2023) and informed by Stockham and Scott (2013)

Physiological grouping	Analyte (abbreviation) (unit)	Brief interpretation	Notes
Metabolic	Albumin (ALB) (g/dl)	Increases with dehydration; decreases with some liver or renal disorders	
Metabolic	Alkaline phosphatase (ALP) (U/I)	Increases with some liver and bone disorders; increases during bone growth	
Metabolic	Alanine aminotransferase (ALT) (U/I)	Increases with some liver and muscle disorders	
Metabolic	Calcium (CA) (mg/dl)	Tightly regulated; important for enzymatic, muscular and neurologic function	
Metabolic (when low) and immune (when high)	Globulin (GLOB) (g/dl)	Decreases with malnutrition and some liver or renal disorders; increases with immune response (antibodies)	Calculated as total protein (TP) – ALB. TP not included in index to avoid collinearity.
Metabolic	Sodium (NA) (mmol/l)	Osmoregulation; regulated by hormonal systems and renal function	
Metabolic (for the purpose of this study)	Packed cell volume (PCV) (%)	Increases with dehydration	
Metabolic	Phosphorus (PHOS) (mg/dl)	Tightly regulated; increases with decreased renal elimination, imbalances with malnutrition or bone disorders	
Metabolic	Potassium (POT) (mmol/l)	Tightly regulated by hormonal systems and renal function	
Metabolic	Urea–creatinine ratio (UC ratio)	Decreases in a fasting state	Short-term fasting biomarker; lower values (≤10.0) indicate fasting over at least 10–14 days (Nelson *et al.,* 1984, Derocher *et al.,* 1990). Calculated as serum urea divided by creatinine (CREA). Serum urea is blood urea nitrogen (BUN)/0.466 (Nelson *et al.,* 1984).
Immune	Basophil count (BAS) (count per μl)	Increases in innate immune response	
Immune	Eosinophil count (EOS) (count per μl)	Increases in response to parasitic infection	
Immune	Lymphocyte count (LYM) (count per μl)	Increases with adaptive immune response	We evaluated the neutrophil–lymphocyte ratio (NLR), a biomarker of acute to chronic immune stress, for inclusion in the model; however, NLR exhibited less variation than NEU and LYM separately.
Immune	Monocyte count (MON) (count per μl)	Increases with immune response including viral infections and chronic conditions	
Immune	Neutrophil count (NEU) (count per μl)	Increases in response to bacterial or fungal infection	
Neuroendocrine	Hair cortisol concentration (HCC) (pg/mg)	Elevated or depressed values can indicate chronic stress (see Edes *et al.,* 2016)	HCC is to be interpreted for year *t* – 1, since cortisol is presumably deposited in polar bear hair during the hair growth period of May–October of the prior year (see Van der Walt *et al.,* 2021).

We calculated *z*-scores for analyte values within demographic groups, which included 1) spring-captured adult females with 1- or 2-year old cubs (lactating), 2) spring-captured adult females without cubs (non-lactating), 3) spring-captured adult males, 4) spring-captured sub-adult females and 5) spring-captured sub-adult males. Adults were bears ≥5 years old and sub-adults were aged 3–4 years (Fry *et al.,* 2019). Only bears having data for >50% of all analytes were retained in analyses (Supplementary Table 1). Although the allostatic load index was calculated using analyte data from bears captured between 1983 and 2018, the final dataset only included bears captured from 1983 to 2016, due to the lack of analyte data in 2017 and the fact that bears captured in 2018 had <50% of the required analytes to be retained in analyses.

### Classifying potential dysregulation via the sample distribution method and aggregating allostatic load

We used a sample distribution method to classify potential dysregulation (following Seeman *et al.,* 1997, Edes *et al.,* 2016). Analytes were split into quartiles or octiles within each demographic group. We defined potential dysregulation as analyte values that were less than the 12.5% octile or greater than the 87.5% octile for ALB, CA, NA, PHOS, POT and HCC (Table 1); a two-tailed approach can be beneficial for classifying potential dysregulation for some markers (Seplaki *et al.,* 2005). We defined potential dysregulation when values were greater than the 75% quartile for PCV, ALP and ALT, and BAS, EOS, LYM, MON and NEU. The UC ratio was classified as at potential dysregulation when values were less than the 25% quartile, indicative of a bear in a fasting state. GLOB was defined as at potential dysregulation when values were less than the 12.5% octile, representing a possible indicator of hepatic or renal issues, or greater than the 87.5% octile, as this may indicate immune activity and greater antibody production.

The allostatic load per individual was calculated as the number of analytes indicating potential dysregulation divided by the total number of analytes available for the individual (maximum 16 analytes available; Table 1). Thus, the index reflects cumulative potential physiological dysregulation and ranges between 0 (no analytes measured are classified as being potentially dysregulated) and 1 (all analytes measured are potentially in dysregulation). Any analyte for which an individual did not have data was not counted towards the denominator of the index and thus the allostatic load for an individual was not influenced by missing data.

### Identifying a health outcome

We examined allostatic load in relation to a known health outcome for polar bears. The most reliable metric of health and fitness for polar bears is body mass or body condition (Rode *et al.,* 2020) as higher body mass is correlated with reproductive success and survival (Derocher and Stirling, 1994, 1996). We used linear models to examine allostatic load (predictor variable) in relation to two response variables indicative of condition (standardized per demographic group): 1) body mass (in kilogrammes), and 2) body condition index (BCI). BCI for polar bears is calculated from the residuals of the regression line between mass and length:


$$ BCI=\frac{10.76+\ln (TBM)-3.07\ast \ln (SLBL)}{0.17+0.009\ast \ln (SLBL)} $$

where *TBM* is total body mass (in kilogrammes), *SLBL* is straight-line body length (in centimetres), and parameters are standard values estimated by Cattet *et al.,* 2002 (see Equation 13). We performed these analyses using the full time series of allostatic load data (1983–2016). We hypothesized that increased physiological stress is associated with poor health, and so we predicted that we would see higher allostatic load in bears with lower mass or BCI.

### Onshore versus offshore behaviour

Since 2004, some SB bears have increasingly come onshore during the summer and early fall to forage on bowhead whale (*Balaena mysticetus*) carcasses that remain on land from subsistence harvests. While SB bears that congregate on land to feed on whale carcasses might experience nutritional benefits (Rode *et al.,* 2022), they are at increased risk of exposure to pathogens and human disturbance and conflict (Atwood *et al.,* 2016a, 2017). We used linear models to examine how allostatic load (response variable) differs by summer habitat use (predictor variable), i.e. onshore land use or offshore sea ice use. Following Fry *et al.* (2023), we classified bears as onshore during the previous summer if they had >5% bowhead whale in their diet, as determined by fatty acid analyses of adipose tissue (Atwood *et al.,* 2017; McKinney *et al.,* 2017). We hypothesized that onshore bears have a greater array of stressors than bears that remain on the sea ice year-round, and so we predicted that we would see higher allostatic load in onshore bears. This analysis included bears sampled during 2004–16 given that the onshore behaviour developed at the start of this period.

### Age

We used linear models to examine how age (predictor variable) affects allostatic load (response variable) for adult females and adult males using the full time series of data (1983–2016). We did not model allostatic load by age for sub-adults because this demographic group only included bears aged 3 or 4 years. We evaluated two competing hypotheses regarding age-related differences in allostatic load. One hypothesis posits that older bears have increased physiological stress, consistent with previous studies in humans and other wildlife species, which have found higher allostatic load in older individuals (see Edes *et al.,* 2018). This hypothesis would be supported by a positive relationship between allostatic load and age. An alternative hypothesis posits that younger bears are more vulnerable to physiological stress due to inexperience or the consequences of resource competition, which would be supported by decreasing allostatic load with age.

### Trend in allostatic load over time

We used linear models to examine how allostatic load (response variable) has changed over time (‘year’ as the continuous predictor variable). We hypothesized that long-term environmental changes in the Arctic have caused greater physiological dysregulation for polar bears, and thus we predicted that allostatic load has increased over time (1983–2016) in response to environmental changes.

### Comparing the sample distribution method to a reference interval method

We compared the sample distribution method, described above, for calculating potential dysregulation to a reference interval method, in which potential dysregulation was classified as falling outside of reference intervals specific to SB bear demographic groups (Fry *et al.,* 2019). We used the demographic-specific reference intervals for ALB, ALP, ALT, CA, GLOB, NA, PHOS and POT; see Tables 3 and 4 in Fry *et al.* (2019) for intervals. Due to there not being reference intervals for BAS, EOS, HCC, LYM, MON, NEU, PCV and UC ratio, we retained the sample distribution method for those analytes. For the reference interval method, we used the same demographic groups used in Fry *et al.* (2019), which included spring-captured adult males, spring-captured sub-adult males and spring-captured sub-adult females (all of which were used in the sample distribution method). We also used two demographic groups used in Fry *et al.* (2019) that were not included in our sample distribution method: spring-captured adult females that denned the prior winter (i.e. adult females with cubs-of-the-year) and spring-captured adult females that did not den the prior winter (i.e. adult females with yearling or 2-year old cubs, or lone adult females; Supplementary Table 2). Lone adult females known to have experienced litter loss (i.e. denned and lost their cub(s) before sampling; Rode, 2018) were not included in analyses. We repeated the analyses in which we examined body mass or BCI (response variable) in relation to allostatic load (predictor variable) calculated using the reference interval method.

**Figure 1:**
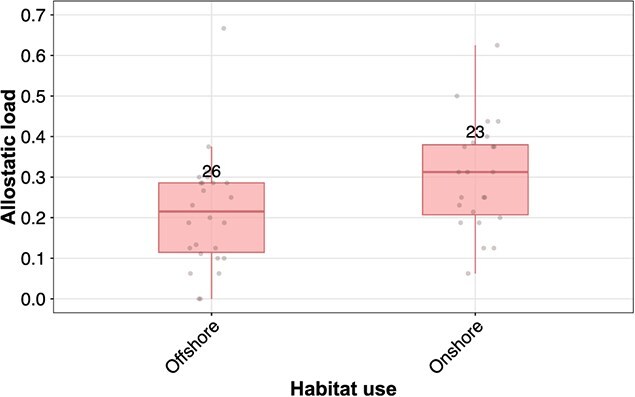
Allostatic load for adult female polar bears without cubs that used offshore habitat (mean allostatic load = 0.21) and those that used onshore habitat (mean = 0.30) during the prior year’s summer and fall sea-ice break up, spanning from 2004 to 2016. Sample size is above each boxplot.

### Examining changes in allostatic load for recaptured and resampled bears

We performed a longitudinal analysis using data from recaptured adult bears (Supplementary Table 3). First, we plotted the change in allostatic load (calculated via the sample distribution method) over time for resampled individuals. Then, we examined the average change in allostatic load for resampled individuals, binned into 5-year intervals between resamples. For bears sampled more than twice, we only considered the change in allostatic load between the earliest and latest capture. We report the mean and 95% confidence interval (CI) change in allostatic load based on the interval.

### Sensitivity analysis to assess the relative impact of each analyte on allostatic load

We performed a *post hoc* sensitivity analysis by systematically removing each analyte and recalculating allostatic load for every bear without the removed analyte. For each scenario, we report the mean absolute change in allostatic load and 95% CI. This analysis focused on adult females without cubs and used the sample distribution method to calculate allostatic load.

### Implementation and model checking

We used R version 4.3.0 (R Core Team, 2023) and fit linear models using the ‘lm’ function in base R. We evaluated model assumptions of linearity, normality and homoscedasticity; and we examined hat values and Cook’s distances to detect high influence points. Though there were multiple samples from some individuals, we did not include a random effect of individual in the linear model analyses (a decision supported by model diagnostics). We report linear model parameter estimates, standard errors, and bootstrapped CIs (95%) for parameter estimates. We generated 100 bootstrapped realizations of the dataset to predict the expected change in body mass or BCI as a function of allostatic load.

## Results

### Applying the allostatic load indices using the sample distribution method

- *Allostatic load in relation to body mass and body condition index*

We found no relationship between allostatic load and body mass (standardized) or BCI (standardized) for any demographic group (Table 2). Model coefficients for allostatic load in all models had 95% CIs that overlapped zero, indicating no evidence of a linear relationship between allostatic load and body mass or BCI.

- *Allostatic load in relation to onshore and offshore habitat use*

**Table 2:** The effect of allostatic load on body mass (BM) (in kilogrammes; standardized) or body condition index (BCI) (standardized; derived from Cattet *et al.,* 2002) for southern Beaufort Sea polar bear demographic groups captured in spring between 1983 and 2016. All models had 95% CIs that overlapped zero

Demographic group	Response variable	*n*	β	SE	95% CI
Adult female with 1- or 2-year old cubs	BM	80	0.539	0.706	−0.866, 1.944
Adult female with 1- or 2-year old cubs	BCI	72	0.612	0.752	−0.888, 2.113
Adult female without cubs	BM	230	−0.037	0.472	−0.967, 0.894
Adult female without cubs	BCI	165	0.870	0.539	−0.194, 1.934
Adult male	BM	203	0.163	0.479	−0.782, 1.107
Adult male	BCI	196	0.283	0.489	−0.681, 1.247
Sub-adult female	BM	49	−0.801	1.004	−2.821, 1.219
Sub-adult female	BCI	46	−0.252	1.230	−2.730, 2.226
Sub-adult male	BM	42	−0.768	0.976	−2.740, 1.204
Sub-adult male	BCI	41	−1.211	1.107	−3.449, 1.027

For adult female bears without cubs between 2004 and 2016, onshore habitat use during the previous summer and fall was associated with greater allostatic load (β_onshore_ = 0.088, 95% CI [0.009, 0.167], Table 3, Fig. 1). The mean allostatic load for adult females without cubs that used onshore habitats the prior summer and fall (*n* = 23) was 0.30; the mean allostatic load for those that remained offshore (*n* = 26) was 0.21 (Fig. 1). For the other demographic groups, we did not detect a difference in allostatic load as a function of habitat use (Table 3).

- *Allostatic load in relation to age*

**Table 3:** The effect of onshore habitat use on allostatic load for southern Beaufort Sea polar bear demographic groups between 2004 and 2016. Onshore behaviour is coded as 1 and offshore behaviour is coded as 0, such that a positive coefficient is indicative of higher allostatic load in bears using onshore habitat. * = 95% CI excluded zero

Demographic group	*n*	β (onshore)	SE	95% CI
Adult female with 1- or 2-year old cubs	26	−0.084	0.058	−0.202, 0.035
Adult female without cubs	49	0.088	0.039	0.009, 0.167*
Adult male	105	0.018	0.028	−0.038, 0.074
Sub-adult female	17	0.048	0.075	−0.111, 0.207
Sub-adult male	13	−0.050	0.067	−0.198, 0.098

Allostatic load declined with increasing age for adult females without cubs (*n* = 228, β = −0.0036, 95% CI [−0.0071, −0.0001], age range = 5–29 years [median: 9]). We did not detect a relationship between allostatic load and age for adult females with 1- or 2-year old cubs (*n* = 80, β = 0.011, 95% CI [−0.0065, 0.0087], age range = 5–25 years [median: 13]), or for adult males (*n* = 202, β = 0.0008, 95% CI [−0.0036, 0.0052], age range = 5–27 years [median: 10]).

- *Temporal trends in allostatic load*

**Figure 2:**
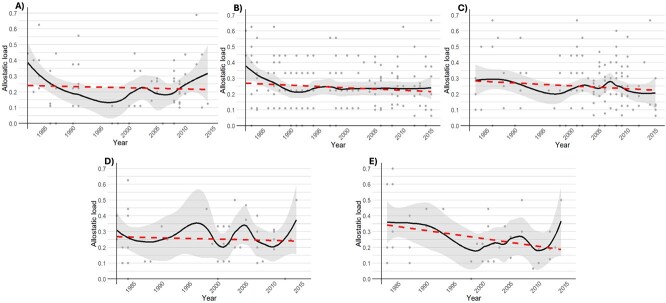
Allostatic load plotted over time for the following southern Beaufort Sea polar bear demographic groups between 1983 and 2016: A) adult females with 1- or 2-year old cubs (*n* = 80), B) adult females without cubs (*n* = 230), C) adult males (*n* = 203), D) sub-adult females (*n* = 49) and E) sub-adult males (*n* = 42). The dashed lines are the linear regression lines. The solid lines are LOESS (locally estimated scatterplot smoothing) lines with ‘span’ (the amount of smoothing) set to 0.5. The shaded portions are the 95% CIs around the LOESS lines. Dots are data points. Allostatic load decreased over time for sub-adult males (β = −0.0050, 95% CI [−0.0099, −0.0001]). There was no linear change in allostatic load for adult females, adult males or sub-adult females.

We did not detect a trend in allostatic load over the period 1983–2016 for lactating adult females (β = −0.0016, 95% CI [−0.0051, 0.0020]), non-lactating adult females (β = −0.0016, 95% CI [−0.0036, 0.0004]), adult males (β = −0.0018, 95% CI [−0.0046, 0.0009]), or sub-adult females (β = −0.0009, 95% CI [−0.0049, 0.0032]) (Fig. 2, Table 4). However, allostatic load decreased over the period for sub-adult males (β = −0.0050, 95% CI [−0.0099, −0.0001]) (Fig. 2, Table 4). Allostatic load was not different across demographic groups (F = 0.27, *P* = 0.90).

**Table 4:** Linear trend estimates for allostatic load (response variable) over the period 1983–2016 (predictor variable) for southern Beaufort Sea polar bear demographic groups. * = 95% CI excluded zero

Demographic group	*n*	Allostatic load range (mean)	β	SE	95% CI
Adult female with 1- or 2-year old cubs	80	0.00–0.75 (0.24)	−0.0016	0.0018	−0.0051, 0.0020
Adult female without cubs	230	0.00–0.67 (0.24)	−0.0016	0.0010	−0.0036, 0.0004
Adult male	203	0.00–0.67 (0.25)	−0.0018	0.0014	−0.0046, 0.0009
Sub-adult female	49	0.00–0.63 (0.25)	−0.0009	0.0020	−0.0049, 0.0032
Sub-adult male	42	0.00–0.70 (0.26)	−0.0050	0.0024	−0.0099, −0.0001*

### Comparing the sample distribution method to the reference interval method

**Figure 3:**
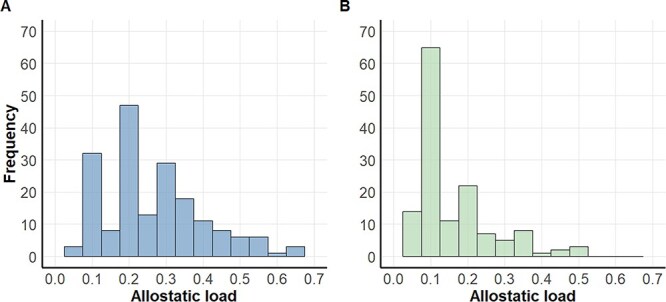
Allostatic load estimates for adult male polar bears calculated using the sample distribution method (panel A; *n* = 203, mean allostatic load = 0.25) versus the reference interval method (panel B; *n* = 203, mean = 0.12).

The reference interval method resulted in allostatic load scores that were lower than those produced from the sample distribution method for matched demographic groups. For instance, the mean allostatic load for adult males using the sample distribution method was 0.25, while the mean allostatic load using the reference interval method was 0.12 (Fig. 3). Sub-adult males and sub-adult females had on average 0.11 and 0.10 lower allostatic load scores, respectively, with the reference interval method. Still, using reference intervals made few differences to the results of the body mass/BCI analyses. All 95% CIs included zero, except for an adult male model in which the reference interval method resulted in a positive estimated relationship between standardized mass and allostatic load (β = 1.162, 95% CI [0.057, 2.268]).

### Examining changes in allostatic load for recaptured and resampled bears

**Figure 4:**
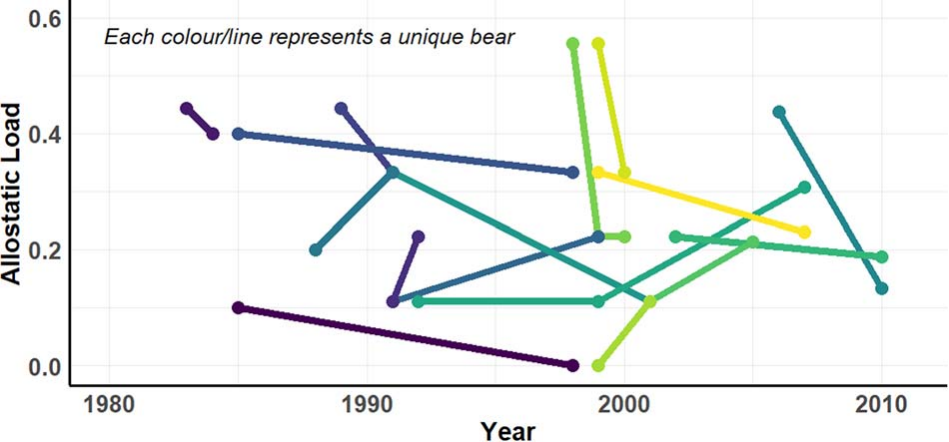
Change in allostatic load over time for recaptured adult female polar bears without cubs (*n* = 16). Each dot represents a sampling time point, and each line and colour combination represent a unique bear.

**Figure 5:**
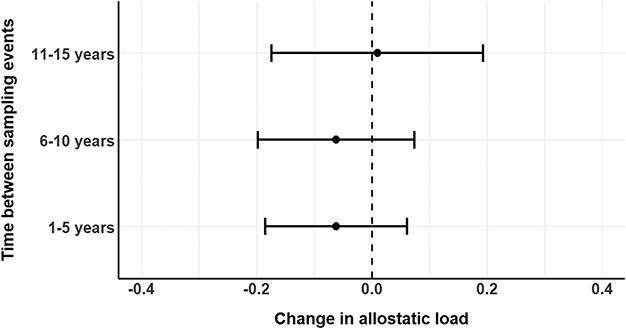
The average change in allostatic load, by length of interval between sampling events, for adult female polar bears without cubs that were sampled multiple times over years in the study (*n* = 16 bears). For bears sampled more than twice, only the first and last samples were retained for this analysis. Points represent means and bars represent 95% CIs.

Sixteen adult female bears without cubs, 5 adult females with 1- or 2-year old cubs, and 26 adult males were sampled multiple times. We were able to detect changes in allostatic load over time for individual bears (Fig. 4). However, when analysing the population, there was no clear pattern of change in allostatic load for any demographic. For adult females without cubs, the mean change in allostatic load was −0.06 for individuals resampled within 1–5 years (*n* = 9, 95% CI [−0.19, 0.06]), −0.06 for individuals resampled within 6–10 years (*n* = 4, 95% CI [−0.20, 0.07]) and 0.01 for bears resampled within 11–15 years (*n* = 3, 95% CI [−0.17, 0.19], Fig. 5). Similarly, for adult females with cubs and adult males, there was no notable change in allostatic load upon resampling and confidence intervals for the mean estimate included zero.

### Sensitivity analysis

**Figure 6:**
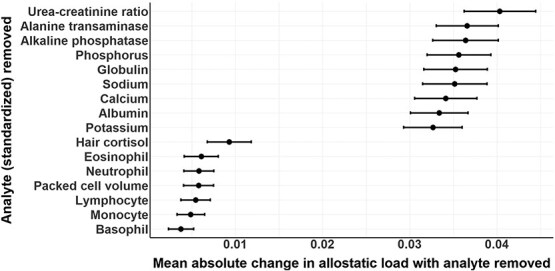
Sensitivity analysis showing the mean absolute change in allostatic load after the sequential removal of each analyte from allostatic load calculations. The analysis was conducted on adult female polar bears without cubs (*n* = 230). Points represent means and bars represent 95% CIs.

Allostatic load calculations were more affected by the removal of the serum metabolic analytes (including UC ratio, ALT, ALP, PHOS, POT, GLOB, NA, CA, ALB, POT) than the blood cell counts (LYM, NEU, MON, BAS, EOS, PCV) or hair cortisol (Fig. 6). Removing the metabolic analytes led to an estimated average absolute change in allostatic load ranging from 0.03 to 0.05 per analyte. In comparison, the removal of hair cortisol and blood cell counts resulted in smaller average absolute changes of ~0.01 and <0.01 per analyte, respectively.

## Discussion

Developing an allostatic load index for any species requires careful consideration of physiological and ecological context, appropriate component health indicators, definitions of potential for dysregulation and a thoughtful approach to modelling the resultant indices. Allostatic load indices are cumulative in nature and incorporate multiple measures of metabolic, immune and neuroendocrine stress and functioning. We selected analytes that have high relevance for understanding polar bear health, given the challenges they face. Our study yielded mixed findings, highlighting the complex nature of ascribing potential physiological dysregulation, and selecting appropriate health indicators. We were unable to detect a relationship between allostatic load and health outcomes, except for adult males using the reference interval method of calculating allostatic load only, where the relationship was contrary to our prediction; specifically, we found a positive relationship between potential physiological dysregulation and mass. We observed that allostatic load declined with age in adult females without cubs, potentially reflecting greater stress experienced by younger, inexperienced individuals, although we did not observe a similar trend in adult females with cubs, nor in adult males. Additionally, we did not observe a trend in allostatic load over time for most demographic groups, although one finding contradicted our predictions: specifically, we found a negative trend in allostatic load over time for sub-adult males. Our prediction regarding the relationship between habitat use and allostatic load was supported only for adult females without cubs. We detected changes in allostatic load over time for individual bears that were repeatedly sampled, although no clear pattern emerged within demographic groups, suggesting that allostatic load may be more effectively interpreted on an individual monitoring basis. Overall, our results prompt further evaluation of underlying assumptions and mechanisms involved with the allostatic load index for polar bears.

We acknowledge a key limitation of allostatic load studies: the assumption that the index represents ‘dysregulation’, even though the markers used to measure allostatic load reflect normal, adaptive physiological responses to stressors. These markers may vary considerably between individuals, fluctuate with circadian rhythms and have multi-system origins and targets, meaning that allostatic load may not accurately pinpoint which process is affected. It is even argued that in wild populations, chronic stress-induced ‘dysregulation’ does not occur, as dysregulation would suggest that the system is malfunctioning when it is actually functioning adaptively to increase fitness in response to challenges (Boonstra, 2012). For example, cortisol, often interpreted as a marker of maladaptive stress, is necessary to overcome a threat by maintaining blood pressure and metabolic processes, among other functions. Thus, the underlying assumptions of allostatic load may limit its broader applicability. Nevertheless, applying this framework to SB polar bears was valuable both theoretically and for its conservation relevance.

Identifying an appropriate health outcome or disease to use as a response variable in an allostatic load model for polar bears was difficult. Body mass or BCI may not be ideal measures, given how quickly polar bears can gain or lose mass based on season and food availability. We considered alopecia syndrome (Atwood *et al.,* 2015, Bowen *et al.,* 2015) as a health outcome. However, while alopecia has been recorded in the SB sub-population, it occurs at low prevalence, with only 49 cases documented from 1998 to 2012 (prevalence: 3.45%, 49 of *n* = 1421 bears sampled; Atwood *et al.,* 2015), and thus we concluded (a conclusion supported by preliminary analyses) that it was too rare to be useful as a health outcome for evaluating our allostatic load index. While the lack of cause-specific mortality data complicates examining mortality as a health outcome, it would be possible to evaluate the effect of allostatic load, perhaps by age group, on survival using a mark–recapture model. Mark–recapture models including temporally varying individual covariates (which are challenging because the covariate cannot be observed when the individual is not observed) have been described (Bonner *et al.,* 2010), though such models are technically complex and data-hungry. The construction of such models could be a fruitful avenue for future research.

The classification of potential physiological dysregulation is important for model performance; results should be interpreted in recognition of the challenges associated with determining what values of an analyte may constitute dysregulation. The optimal method for classifying potential physiological dysregulation and constructing an allostatic load index is debated (McLoughlin *et al.,* 2020). Indices may include thresholds that represent early risk for morbidity or mortality, and/or those that are actively in dysregulation. Edes and Crews (2017) argue that sub-clinical risk is best measured using a sample distribution method as opposed to clinical reference interval cut points, as the latter only retains analytes that are already in a state of dysregulation and ignores early risk. We applied both the sample distribution and reference interval method to the body mass and BCI analyses and found overall minimal differences between the two. An alternative method to quantify physiological dysregulation is an epigenetic-based measure of biological age, which represents accumulated physiological damage over time.

We found partial support for the hypothesis that allostatic load declines with age, as this was only evident for one of the three demographic groups tested—adult females without cubs. Another hypothesis that deserves more exploration is that the relationship between allostatic load and age is non-linear—for instance, with allostatic load peaking in young, inexperienced adults and again in older adults, although we did not observe this in the data. Alternatively, the fact that we had relatively few observations of old females may have diminished our power for detecting an effect. This may have been partly due to older individuals dying and being removed from analysis.

Analytes or biomarkers relating to hydration, liver or kidney function, neuroendocrine stress and immune function are important for understanding the health of polar bears (Fry *et al.,* 2019, 2023; Robbins *et al.,* 2021; Van der Walt *et al.,* 2021). Fry *et al.* (2023) observed increasing levels of ALT in SB bears from 1984 to 2018, suggesting potential liver cell (hepatocyte) damage with environmental change and shifts in prey base. Polar bears derive water from metabolizing dietary blubber from their marine mammal prey (Nelson *et al.,* 1983); thus, reduced availability of prey, decreased fatness of prey or overconsumption of protein could increase water imbalance (Fry *et al.,* 2023) and renal or hepatic disease (Robbins *et al.,* 2021). Our sensitivity analysis revealed that metabolic markers (e.g. UC ratio, ALT, ALP) had more influence on allostatic load than immune cell counts or hair cortisol. However, the impact of individual metabolic analytes was relatively mild. The signal of physiological dysregulation can be robust to the choice of component analytes, since dysregulation is often multi-systemic (Cohen *et al.,* 2015). While some studies assign different weights to specific analytes (see Edes *et al.,* 2018), we elected to use equal weighting for all analytes, given this is the first comprehensive study of allostatic load in polar bears. An additional consideration is a negative feedback effect occurring with interacting analytes, where the elevation of one analyte results in the suppression of another; this type of effect may not be captured in our allostatic load index. Further, the samples used reflect analyte values that reference different temporal windows. HCC accumulates during hair growth (summer and fall) and blood-based analytes reference a couple of weeks to a month prior to collection.

Many allostatic load indices take a data reductionist approach, e.g. scoring analytes for dysregulation and using the scores in the model, or clustering the data to decrease dimensionality (e.g. principal components analysis or latent factor analysis). The risk of reducing the data is the potential to obscure patterns, while the benefits are the ability to explicitly measure dysregulation (as opposed to innate variation) and the ability to use a common linear modelling approach for all analytes. Another consideration when designing allostatic load indices is that much of this framework is circular: physiological dysregulation causes disease, which causes further dysregulation. A future study could explore a modelling approach that attempts to account for this circularity, such as structural equation modelling (Pogiotta *et al*., 2025).

While the concept of allostatic load has gained traction in recent years, there are alternative health scoring indices that have been used to predict health outcomes for other wildlife species. For tundra caribou (*Rangifer tarandus*), scientists developed a health framework that incorporated 1) health determinants (e.g. exposure to pathogens and trace elements), 2) health processes (i.e. physiological responses to health determinants, e.g. stress hormones or pathology) and 3) health outcomes (e.g. body condition or pregnancy; Aguilar *et al*., 2023). For bottlenose dolphins (*Tursiops truncatus*), a weighted health scoring system was used to evaluate blood parameters, where individuals scored more points based on increasing deviation from a normal range (Wells *et al.,* 2004). In another study with bottlenose dolphins, veterinarians and scientists created a health index that consisted of signs of inflammatory, metabolic, pulmonary and neuroendocrine functioning and related this to survival probabilities (Schwacke *et al.,* 2023). For North Atlantic right whales (*Eubalaena glacialis*), a hierarchical Bayesian model related individual health status, based on visual data on body and skin condition, to reproductive output (Rolland *et al.,* 2016). These examples of health scoring systems are like allostatic load indices in that they integrate multiple indicators of health. One difference is that allostatic load indices may use wider thresholds to classify potential dysregulation, to account for early signs.

Identifying a method to approximate individual bear health and states of physiological functioning would benefit both research and management. While it is difficult to define and operationalize the concept of health, Patyk *et al.* (2015) put forth a definition for polar bear health, stating that health can be applied at the ‘individual, species and ecosystem levels’ and is determined by whether a population ‘can respond to factors in its environment and sustain itself in the long term’. Gaining a better understanding of individual health and physiology could allow scientists to link individual health profiles to outcomes such as disease (Patyk *et al.,* 2015) and predict the probability that an individual could transition into a state of poor health based on physiological measures. These predictions could be linked to population vital rates, such as survival, fecundity and population growth. For a sub-population at risk of decline, like the SB, such attention to individual health may be useful for conservation (Patyk *et al.,* 2015). The methods we present could serve as a tool for future studies seeking to apply and improve the allostatic load index.

## Supplementary Material

Web_Material_coaf013

## Data Availability

The data used in these analyses were obtained from Atwood, 2023 (https://doi.org/10.5066/P9OXCRJ6), Durner, 2021 (https://doi.org/10.5066/P9RP5KJP), Rode, 2018 (https://doi.org/10.5066/F7DF6PC9), and Rode, 2020 (https://doi.org/10.5066/P9TVK3PX). These datasets are publicly available at the DOI links provided.
