# Supplementary material for: Measuring polar bear health using allostatic load
Source: Conserv Physiol. 2025 Mar 5;13(1):coaf013. doi: 10.1093/conphys/coaf013 (PMC11884737; doi:10.1093/conphys/coaf013)
Supplement: Web_Material_coaf013 [file web_material_coaf013.zip › Supplementary File 1.pdf]

Supplementary File 1

Table S1. Sample size of Southern Beaufort Sea polar bears sampled between 1983-2016 and eligible for inclusion in analyses where allostatic load was calculated using the sample distribution method. Eligibility for inclusion is based on the criteria of a bear having data for more than 50% of the analytes comprising the allostatic load index. Inclusion in specific analyses depends on the data requirements for each analysis. For example, a bear with data for more than 50% of the analytes but lacking mass data would be excluded from the mass analysis but could still be included in the onshore/offshore analysis if such data were available. The actual sample sizes are provided in the relevant sections of the paper.

	Adult females without cubs (non-lactating)	Adult females with one- or two-year old cubs (lactating)	Adult males	Subadult females	Subadult males
**Total N**	**230**	**80**	**203**	**49**	**42**
Year					
1983	3	2	0	3	0
1984	9	2	6	2	3
1985	11	5	2	6	4
1986	0	0	0	0	0
1987	5	6	3	0	0
1988	8	3	1	1	3
1989	5	0	3	1	0
1990	8	0	0	0	0
1991	16	6	1	2	2
1992	14	8	7	1	0
1993	7	0	1	0	0
1994	9	0	0	0	1
1995	20	0	0	0	0
1996	0	0	0	0	0
1997	0	0	1	0	0
1998	8	2	1	0	0
1999	14	1	10	1	2
2000	4	0	1	1	1
2001	11	3	15	3	2
2002	3	4	11	4	3
2003	5	4	19	3	4
2004	1	0	1	3	1
2005	5	3	10	1	1
2006	7	3	8	2	3
2007	10	1	12	0	0
2008	0	0	7	6	3

2009	7	8	34	1	0
2010	9	8	19	0	1
2011	4	6	11	5	2
2012	0	0	1	0	0
2013	8	2	8	1	4
2014	4	2	1	1	1
2015	6	1	3	1	1
2016	9	0	6	0	0

Table S2. Sample size of Southern Beaufort Sea polar bears sampled between 1983-2016 and available for inclusion in the analysis where allostatic load was calculated using the clinical reference interval method. Eligibility for inclusion is based on the criteria of a bear having data for more than 50% of the analytes comprising the allostatic load index.

	Recently denned females (females with cubs-of-the-year)	Non-recently denned females (females with one- or two-year old cubs, or solitary females)	Adult males	Subadult females	Subadult males
**Total N**	**87**	**313**	**203**	**49**	**42**
Year					
1983	0	5	0	3	0
1984	5	11	6	2	3
1985	1	16	2	6	4
1986	0	0	0	0	0
1987	2	11	3	0	0
1988	1	11	1	1	3
1989	0	5	3	1	0
1990	1	8	0	0	0
1991	2	22	1	2	2
1992	7	22	7	1	0
1993	1	7	1	0	0
1994	0	9	0	0	1
1995	0	20	0	0	0
1996	0	0	0	0	0
1997	0	0	1	0	0
1998	2	10	1	0	0
1999	4	15	10	1	2
2000	8	4	1	1	1
2001	3	14	15	3	2
2002	5	7	11	4	3
2003	7	9	19	3	4
2004	0	2	1	3	1
2005	5	8	10	1	1

2006	1	10	8	2	3
2007	4	11	12	0	0
2008	3	0	7	6	3
2009	14	15	34	1	0
2010	7	17	19	0	1
2011	0	10	11	5	2
2012	0	0	1	0	0
2013	1	10	8	1	4
2014	1	7	1	1	1
2015	2	8	3	1	1
2016	0	9	6	0	0

Table S3. Number of adult Southern Beaufort Sea polar bears included in allostatic load analyses that were recaptured and resampled throughout the study period (1983-2016).

Demographic	Total sampled >2 times	Sampled twice	Sampled three times	Sampled four times
Adult females without cubs (non-lactating)	16	14	2	0
Adult females with one- or two-year old cubs (lactating)	5	4	1	0
Adult males	26	16	8	2
